# Surprise billing in intensive care unit (ICU) hospitalizations

**DOI:** 10.1093/haschl/qxae025

**Published:** 2024-02-27

**Authors:** Sneha Kannan, Zirui Song

**Affiliations:** Division of Pulmonary/Critical Care, Massachusetts General Hospital, Boston, MA 02114, United States; Department of Health Care Policy, Harvard Medical School, Boston, MA 02115, United States; Department of Health Care Policy, Harvard Medical School, Boston, MA 02115, United States; Department of Medicine, Massachusetts General Hospital, Boston, MA 02114, United States

**Keywords:** surprise billing, balance billing, cost of care, intensive care unit

## Abstract

Intensive care unit (ICU) care is expensive for patients and providers, and utilization and spending on ICU resources have increased. The No Surprises Act, passed in 2022, specifically prohibits balance billing by ICU specialists (intensivists) for emergency and most non-emergency care. The potential economic impact of this remains unclear, given few data exist on the magnitude of balance billing in the ICU. Using the MarketScan Commercial (IBM) database, we studied hospitalizations in which ICU care was provided (“ICU hospitalizations”) between 2010 and 2019. Hospitalizations were characterized as fully in-network, fully out-of-network, or “mixed” (contained both in- and out-of-network services). The share of “mixed” hospitalizations among all ICU hospitalizations rose from 26% to 33% over the study period. Over half of these mixed hospitalizations contained out-of-network services specifically delivered within the ICU. Total hospitalization spending averaged $81 047, with ICU spending averaging $15 799. On average, 11% of ICU spending within these hospitalizations was out-of-network. Patients were plausibly balance-billed in approximately one-third of ICU hospitalizations, for thousands of dollars per hospitalization. Given that the No Surprises Act prevents this type of balance billing, the portended revenue loss may lead to changes in provider negotiations with insurers concerning network status and prices, which could affect the care patients receive.

## Lay Summary

When patients receive out-of-network services, even within an in-network hospitalization, they can receive a “surprise bill,” typically the balance (or difference) between what providers charge and what an insurer pays. This balance can be large, and contributes to the hospital or physician revenue generated from patients. The No Surprises Act, passed in 2022, protected patients from many types of surprise bills (while exempting some) and required providers to collect any extra out-of-network payments beyond plan-specified patient cost-sharing from the insurers in most situations. In particular, the No Surprises Act specified that patients cannot receive a balance bill for care by intensive care unit (ICU) doctors in most situations (exceptions can be made for some patients who receive non-emergency care at out-of-network hospital ICUs). To understand the potential implications of this loss of revenue to hospitals, we studied ICU hospitalizations among patients with employer-sponsored health insurance. We found that approximately one-third of hospitalizations involving ICU care had the potential for balance billing. Insurers spent an average of $80 000 per ICU hospitalization. Several thousands of those dollars were spent on out-of-network services in the ICU, where hospitals likely received substantial additional revenue from patient balance bills. After the No Surprises Act was passed, hospitals are no longer able to receive this revenue, which likely has implications for patients and physician groups.

## Teaser

Intensive care unit (ICU) care is expensive, and an important source of revenue for hospitals. After the No Surprises Act was passed in 2022, ICUs were no longer able to balance bill for services. However, the extent to which ICUs and hospitals may lose revenue as a result of this policy remains unknown.

## Introduction

The No Surprises Act, which went into effect in 2022, aims to protect commercially insured patients from most common forms of “surprise billing” (whereby patients are billed the balance, or difference, between provider charges and insurer payments for care provided by out-of-network clinicians at in-network facilities, care at out-of-network facilities with few exceptions, and out-of-network air ambulances).^1,2^ The law specifically protects against balance billing for services provided in intensive care units (ICUs) in both emergency and non-emergency settings, except in specific situations where ICU care is non-emergent and provided at an out-of-network facility.

ICU services are expensive for payers and patients.^3^ Additionally, patient care in ICUs is typically provided by large, multidisciplinary teams, which frequently include the services of consultants across multiple specialties as well as intensivists (ICU physicians).^4,5^ This structure suggests that ICUs may be prone to provide out-of-network services in otherwise in-network hospitals. Therefore, patients cared for in ICUs may be particularly vulnerable to surprise billing. Furthermore, unlike many other high-priced medical services, ICU services are typically determined by clinician decisions, over which patients usually have little to no input (especially given the acuity of illness for many ICU patients). Since average cost-sharing for ICU hospitalizations is already higher than what many Americans are able to pay in one-time emergency expenses, surprise billing for ICU care would likely more often be prohibitively expensive for patients.^6,7^

To date, however, the potential impact of this ICU provision of the No Surprises Act remains unknown. The reason is that, despite ICUs being a large source of revenue for hospitals, the frequency and magnitude of potential balance billing within hospitalizations involving ICU services (“ICU hospitalizations”) have not been documented. This magnitude may be particularly important economically, as the prohibition of balance billing for ICU care could lower ICU revenue. This, in turn, could affect how hospitals provide ICU care and how providers negotiate with payers over their network status and the prices of ICU care.

As ICU use and spending grow nationally, ICU reimbursement rules are increasingly consequential.^8^ Although the No Surprises Act protects patients from surprise billing for ICU hospitalizations, downstream changes in ICU provider networks, negotiated prices, or the care delivered may affect critically ill patients both clinically and financially. These reverberations likely depend on the extent of ICU-related revenue at stake in the No Surprises Act. Thus, to understand this potential impact of the No Surprises Act, we examined the frequency of out-of-network care, and potential surprise billing, within ICUs.

## Data and methods

We examined adult hospitalizations from 2010 through 2019 in the MarketScan Commercial database (published by IBM), which contains a large, nationwide convenience sample of individuals with employer-sponsored insurance. We excluded 2020 and 2021 because of the COVID-19 pandemic. ICU hospitalizations were identified using revenue codes validated previously.^9^ For each service, we obtained the in- or out-of-network status of its clinician and the paid amount. We categorized services broadly using Current Procedural Terminology (CPT) codes into the categories of Evaluation and Management, Anesthesia, Surgery, Radiology, Laboratory Services, and Other.^10^ We identified hospitalizations as entirely in- or out-of-network, or “mixed” if a portion of services within a hospitalization was out-of-network. For “mixed” hospitalizations, we assessed which out-of-network services were provided within the ICU stay using the dates of service. “Mixed” hospitalizations were used because the data did not identify the network status of the facility. These hospitalizations could capture both care provided at in-network and out-of-network facilities, although we hypothesized that the “mixed” hospitalizations predominantly represented out-of-network services delivered at in-network facilities.

## Results

**Figure 1.**
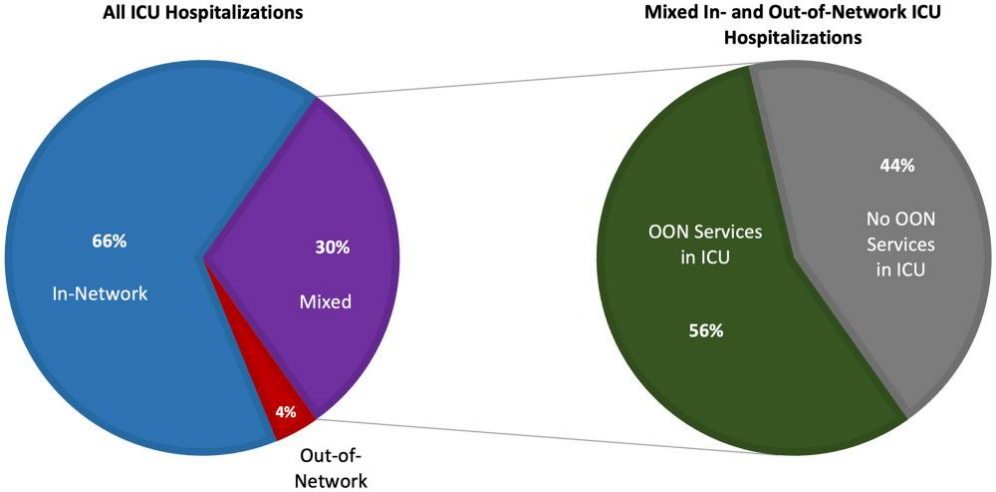
Out-of-network status of ICU hospitalizations. This analysis used adult hospitalizations from 2010–2019 from MarketScan Commercial (IBM) claims data. ICU hospitalizations are defined as hospitalizations where ICU services were provided. This figure decomposes all ICU hospitalizations into entirely in-network, out-of-network, or mixed. Mixed hospitalizations, or those most likely to result in surprise billing, were then decomposed further into hospitalizations where out-of-network services were provided within the ICU. Abbreviations: ICU, intensive care unit; OON, out-of-network.

Within 14 971 584 adult hospitalizations, 1 591 098 contained ICU services. Among these “ICU hospitalizations,” 57 749 (4%) comprised entirely out-of-network services, while 484 012 (30%) were “mixed” ICU hospitalizations that included both in-network and out-of-network services—in which out-of-network clinicians billing at in-network facilities was most likely. From 2010 through 2019, the share of all ICU hospitalizations accounted for by these “mixed” ICU hospitalizations increased from 26% to 33%. More than half of these “mixed” ICU hospitalizations—56% over the study period—contained out-of-network services specifically within the ICU (Figure 1).

Among the sizeable share of “mixed” ICU hospitalizations containing both in- and out-of-network services, a substantial portion of hospitalization spending went to out-of-network services. Total spending among these mixed ICU hospitalizations averaged $81 047, in which ICU spending averaged $15 799. At the hospitalization level, 15% of hospitalization spending was on out-of-network services, on average, and 11% of ICU spending within these hospitalizations was out-of-network. In terms of service categories, surgical services comprised the largest amount of ICU spending ($3079 per hospitalization), of which 25.7% was out-of-network. Evaluation and Management services were the most frequently billed out-of-network category—present in 55% of ICU stays where out-of-network services were billed (Table 1).

**Table 1.** Spending on out-of-network ICU services within hospitalizations.

		ICU hospitalizations with OON ICU services (*n* = 271 046)			
Service category	Share of OON ICU stays (%)	Spending per hospitalization ($)	OON spending as share of hospitalization spending (%)	ICU spending per hospitalization ($)	OON spending as share of ICU spending (%)
E&M	55.2	3426 ± 6977	30.0 ± 34.0	2224 ± 5198	31.6 ± 35.4
Anesthesia	8.0	1285 ± 2653	20.4 ± 20.4	983 ± 2172	20.6 ± 39.4
Surgery	21.2	4071 ± 15 724	25.1 ± 39.4	3079 ± 13 411	25.7 ± 39.9
Radiology	16.8	424 ± 1100	16.2 ± 34.9	274 ± 808	15.9 ± 35
Laboratory	9.6	267 ± 863	20.9 ± 40	172 ± 598	21.2 ± 40.4
Other	30.2	1285 ± 6337	25.5 ± 39.6	775 ± 4542	27.2 ± 41.1

Abbreviations: E&M, Evaluation and Management; ICU, intensive care unit; OON, out-of-network.

Values are means ± standard deviation unless otherwise indicated. This table describes average spending on OON services among those ICU hospitalizations that contained OON services in the ICU. Average hospitalization-level spending and spending on ICU care are shown by broad service category. In addition, the average shares of OON spending within the hospitalization and OON spending within the ICU were calculated at the hospitalization level for each category. Services were classified using Current Procedural Terminology codes. All dollars were adjusted for inflation to 2019 dollars.

## Discussion

Among adults with commercial insurance, 34% of all ICU hospitalizations contained out-of-network services—over half of which contained out-of-network services specifically in the ICU—that plausibly resulted in balance bills. Given that average spending on a hospitalization involving ICU care exceeded $80 000, potential balance billing equates to thousands of dollars per hospitalization.

Prior to the No Surprises Act, out-of-network services likely represented an important and increasingly common source of revenue for ICUs and hospitals, with the variation in our sample suggesting that some hospitals contained out-of-network services more frequently than others. Additionally, hospitals have been increasingly providing care in ICUs. Given that the No Surprises Act forbids balance billing of out-of-network services by intensivists, some clinicians and hospitals likely face a meaningful revenue loss.^11^ While this protects patients from a previously underappreciated source of surprise billing—the ICU, where acutely or severely ill patients typically have little choice or ability to shop as informed consumers—clinicians, staffing firms, and hospitals may, in response, negotiate differently with insurers over their network status and negotiated rates, which could have ripple effects on the care delivered to patients and their out-of-pocket expenses.

Notably, this analysis was limited to enrollees with employer-sponsored insurance and may not be generalizable to other commercially insured populations, including those with Affordable Care Act (ACA) Marketplace or non-group plans. Because our data did not include hospital charges or patient bills, we were not able to directly observe balance billing. However, in line with prior work, we identified encounters where surprise billing was plausible.^12-14^

## Conclusion

Approximately one-third of all ICU hospitalizations contained out-of-network services, and 56% of those hospitalizations contained out-of-network services within the ICU. Balance billing for these out-of-network services has been shown to be an important source of revenue to physicians and hospitals. Given that insurers spent an average of $80 000 per ICU hospitalization, the elimination of balance billing due to the No Surprises Act may result in meaningful revenue losses to providers. Understanding changes in provider–insurer negotiations stemming from this law, notably around network status and negotiated prices, as well as changes in subsequent care delivery and patient out-of-pocket expenses are key areas of future study.

## Supplementary material

Supplementary material is available at *Health Affairs Scholar* online.

## Supplementary Material

qxae025_Supplementary_Data
